# The Mucus Binding Factor Is Not Necessary for *Lacticaseibacillus rhamnosus* CRL1505 to Exert Its Immunomodulatory Activities in Local and Distal Mucosal Sites

**DOI:** 10.3390/ijms232214357

**Published:** 2022-11-18

**Authors:** Kae Tomotsune, Fernanda Raya Tonetti, Hiroya Mizuno, Mariano Elean, Kohtaro Fukuyama, Binghui Zhou, Wakako Ikeda-Ohtsubo, Keita Nishiyama, Akihiro Yamamura, Hideaki Karasawa, Shinobu Ohnuma, Akira Horii, Tadao Saito, Haruki Kitazawa, Julio Villena

**Affiliations:** 1 Food and Feed Immunology Group, Laboratory of Animal Food Function, Graduate School of Agricultural Science, Tohoku University, Sendai 980-8577, Japan; 2 Laboratory of Immunobiotechnology, Reference Centre for Lactobacilli (CERELA-CONICET), San Miguel de Tucuman 4000, Argentina; 3 Livestock Immunology Unit, International Education and Research Center for Food Agricultural Immunology, Graduate School of Agricultural Science, Tohoku University, Sendai 980-8577, Japan; 4 Division of Molecular Pathology, Department of Pathology, Tohoku University Graduate School of Medicine, Sendai 980-8577, Japan

**Keywords:** immunobiotic, mucus-binding factor mutant, *Lacticaseibacillus rhamnsous* CRL1505, intestinal immunity, respiratory immunity, probiotic

## Abstract

Both viable and non-viable orally administered *Lacticaseibacillus rhamnosus* CRL1505 modulate immunity in local (intestine) and distal (respiratory) mucosal sites. So, intestinal adhesion and colonization are not necessary for this probiotic strain to exert its immunomodulatory effects. In this work, a mucus-binding factor knockout CRL1505 strain (Δ*mbf*CRL1505) was obtained and the lack of binding ability to both intestinal epithelial cells and mucin was demonstrated in vitro. In addition, two sets of in vivo experiments in 6-week-old Balb/c mice were performed to evaluate Δ*mbf*CRL1505 immunomodulatory activities. (A) Orally administered Δ*mbf*CRL1505 prior to intraperitoneal injection of the Toll-like receptor 3 (TLR3) agonist poly(I:C) significantly reduced intraepithelial lymphocytes (CD3^+^NK1.1^+^CD8αα^+^) and pro-inflammatory mediators (TNF-α, IL-6 and IL-15) in the intestinal mucosa. (B) Orally administered Δ*mbf*CRL1505 prior to nasal stimulation with poly(I:C) significantly decreased the levels of the biochemical markers of lung tissue damage. In addition, reduced recruitment of neutrophils and levels of pro-inflammatory mediators (TNF-α, IL-6 and IL-8) as well as increased IFN-β and IFN-γ in the respiratory mucosa were observed in Δ*mbf*CRL1505-treated mice when compared to untreated control mice. The immunological changes induced by the Δ*mbf*CRL1505 strain were not different from those observed for the wild-type CRL1505 strain. Although it is generally accepted that the expression of adhesion factors is necessary for immunobiotics to induce their beneficial effects, it was demonstrated here that the *mbf* protein is not required for *L. rhamnosus* CRL1505 to exert its immunomodulatory activities in local and distal mucosal sites. These results are a step forward towards understanding the mechanisms involved in the immunomodulatory capabilities of *L. rhamnosus* CRL1505.

## 1. Introduction

Probiotics with the ability to beneficially modulate the immune system (immunobiotics) have become interesting alternatives to improve the immune health status in both humans and animals and to enhance protection against infections [1]. To be effective, immunobiotics must first go across the physical and chemical barriers of the intestinal mucosa and then be recognized by pattern recognition receptors (PRRs) expressed in intestinal epithelial cells and antigen-presenting cells. Therefore, adhesion to the epithelium has been considered as an essential requirement of immunobiotics for stimulating immunity in the intestinal mucosa [2,3,4].

Among the most studied probiotic strains is *Lacticaseibacillus rhamnosus* GG. This strain has tolerance to gastric acid and bile, so it reaches the intestinal tract alive and adheres to intestinal epithelial cells [1]. Newborn mice whose intestinal tracts were colonized with *L. rhamnosus* GG showed improved maturation in their gut function and increment of IgA production [5]. Those immunomodulatory effects were shown to be partially dependent on the ability of *L. rhamnosus* GG to adhere to the intestinal epithelium [6]. Interestingly, it was also demonstrated that the oral ingestion of non-viable *L. rhamnosus* GG increases interleukin (IL)-6 secretion and IgA production in rats [7], indicating that adhesion is not essential for this strain to exert its beneficial effects. Similarly, our group has extensively studied the immunobiotic strain *L. rhamnosus* CRL1505. We have shown that the oral administration of this immunobiotic strain increases the resistance to intestinal and respiratory infections in immunocompetent and immunosuppressed mice [8,9]. We have also reported that *L. rhamnosus* CRL1505 reduces intestinal and respiratory tract infections in humans [10]. Of note, we showed that the viability of immunobiotic bacteria is not a necessary condition to achieve the immunomodulatory effect [11,12]. 

Genomic analysis of *L. rhamnosus* CRL1505 indicated that among the most important proteins that could be involved on its mucosal adhesion is the mucus-binding factor (*mbf*). This protein contains a repeating structure of 4 Pfam-MucBP (mucin-binding protein) at the N-terminus and a major structural element (LPXTG motif) at the C-terminus [6]. The *mbf* protein is presumed to be an adhesin of the extracellular matrix because the adherence of the *mbf*-deficient *L. rhamnosus* FSMM22 strain to the extracellular matrix was considerably reduced compared to the wild-type (WT) strain [13]. However, reports on the role of *mbf* in the adherence of immunobiotics and their direct effects on the cells of the intestinal tract are scarce. This is probably due to difficulties in synthesizing and chemically purifying bacterial surface proteins with high purity, which delays understanding the relationship between immunomodulatory effects and immune signal receptors in the intestinal tract [4].

The aim of this work was to elucidate whether *L. rhamnosus* CRL1505 preserved its immunomodulatory functions after eliminating the *mbf* protein by means of genetic engineering techniques. For this purpose, we designed a CRL1505 knockout mutant for the *mbf* gene (Δ*mbf*CRL1505 strain), evaluated its adhesion capacity to intestinal epithelial cells and mucins, and assessed the ability of the orally administered mutant strain to modulate the antiviral immune responses in both the intestinal and respiratory tracts of mice, in comparison with the WT CRL1505 strain.

## 2. Results

### 2.1. Construction of L. rhamnosus *Δ*mbfCRL1505

A 600 bp knockout of the CRL1505 *mbf* gene was designed by PCR. This fragment was subcloned in the thermosensitive vector pSG^+^E2 and transformation was performed into *L. lactis* IL1403 competent cells by the electroporation method. The resulting knockout plasmid pSG^+^*mbf* was treated with the restriction enzymes *Sac*I and *Sal*I and confirmed by 1.2% agarose gel electrophoresis where a band corresponding to the insert (1107 bp) and another corresponding to the backbone of the plasmid pSG^+^E2 (3932 bp) were obtained (data not shown).

**Figure 1:**
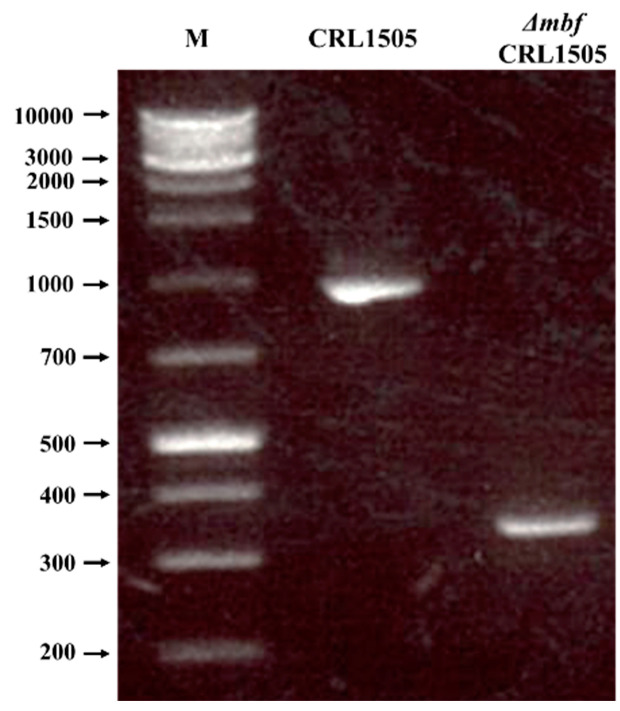
Confirmation of *mucus-binding factor* knockout *Lacticaseibacillus rhamnosus* CRL1505 *(*Δ*mbf*CRL1505) strain obtention by PCR analysis. Control of the double-crossover reaction by 1% agarose gel electrophoresis. M: XL-DNA Ladder 1K plus molecular weight marker (KE 2610—Integrale). CRL1505: PCR of wild-type *L. rhamnosus* CRL1505 genomic DNA using the primers Lr17 and Lr18 (Table 1) as templates. Δ*mbf*CRL1505: PCR of *L. rhamnosus* Δ*mbf*CRL1505 genomic DNA using the primers Lr17 and Lr18 (Table 1) as templates.

Then, the pSG^+^*mbf* plasmid was transformed into *L. rhamnosus* CRL1505 by electroporation and a double-crossover recombination was performed. After isolating the secondary recombinant, genome extraction, PCR and electrophoresis were performed (Figure 1). We then sequenced the amplified gene fragment to confirm that the 600 bp target site was removed from the secondary recombinant. The strain obtained, lacking the sequence of the gene that codes for the mucus-binding factor, was named Δ*mbf*CRL1505.

**Figure 2:**
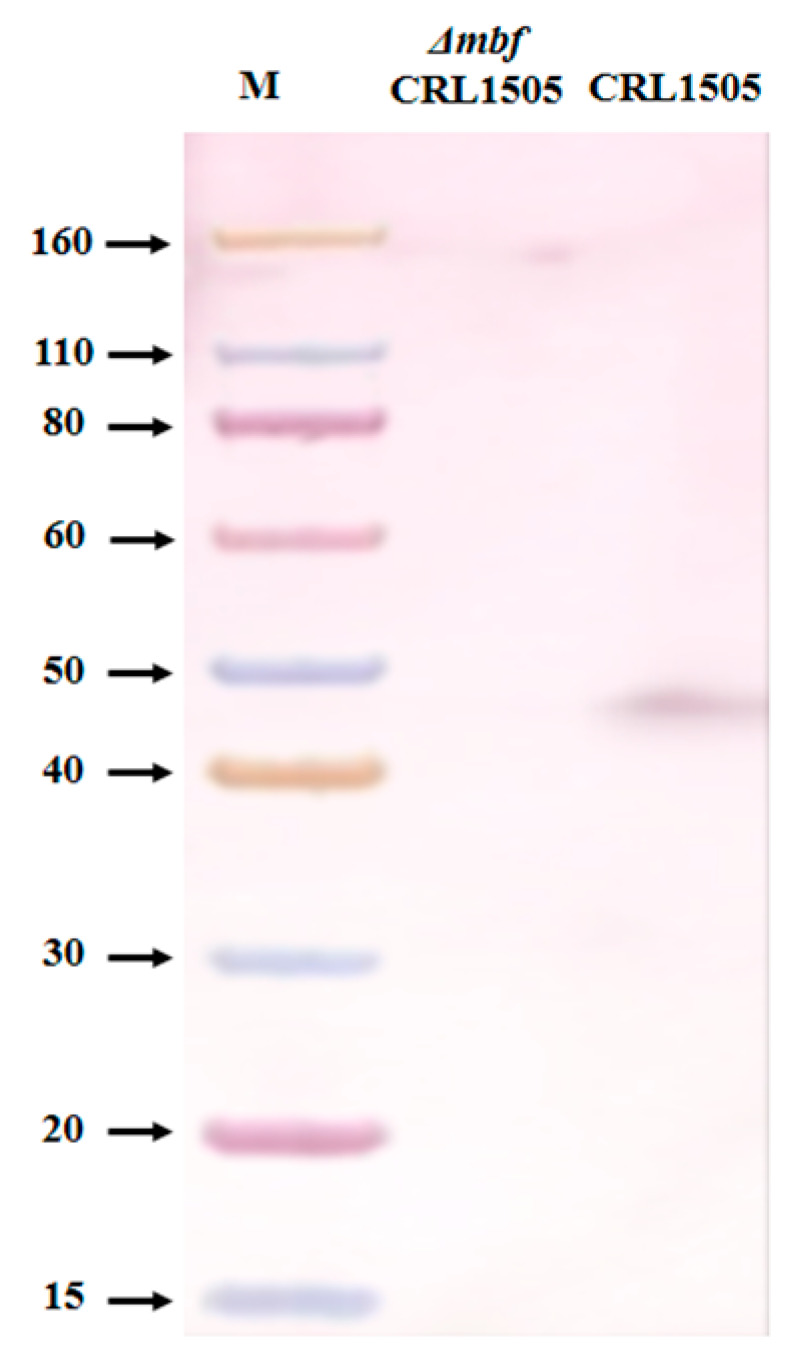
Confirmation of *mucus-binding factor* knockout *Lacticaseibacillus rhamnosus* CRL1505 *(*Δ*mbf*CRL1505) strain obtention by Western Blot analysis. Western blotting using a specific antibody to the *mbf* of *L. rhamnosus* (rabbit anti CRYVRLAADSAAASGTFPKD). M: Protein molecular weight marker (Promega). CRL1505: 45 KDa band belonging to the *mbf* protein of the wild-type *L. rhamnosus* CRL1505 strain. Δ*mbf*CRL1505: Absence of the *mbf* protein in the mutant CRL1505 strain.

Finally, we extracted the surface proteins from the CRL1505 and Δ*mbf*CRL1505 strains and studied the presence of the *mbf* protein by Western blotting using a specific antibody to the *mbf* of *L. rhamnosus* (Figure 2). We observed that the 45 KDa band belonging to the *mbf* protein was present only in the CRL1505 strain but not in Δ*mbf*CRL1505 (Figure 2).

### 2.2. L. rhamnosus *Δ*mbfCRL1505 Phenotypic Evaluation

**Figure 3:**
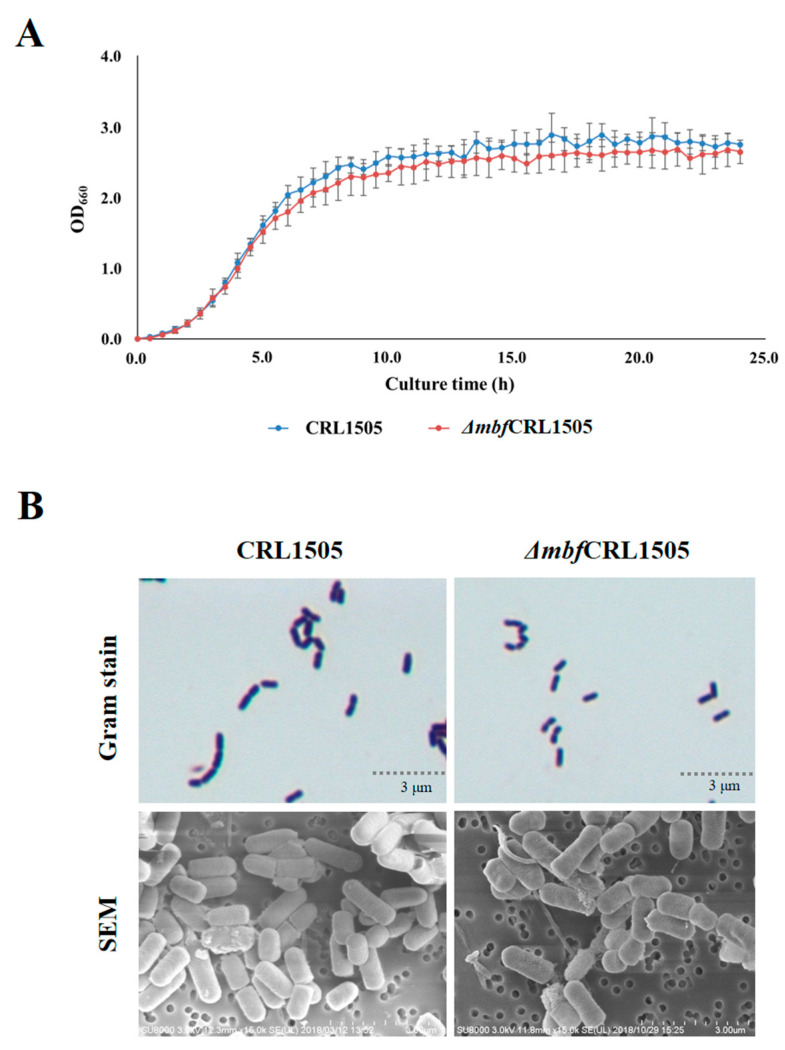
Phenotypic characterization of the *mucus-binding factor* knockout *Lacticaseibacillus rhamnosus* CRL1505 *(*Δ*mbf*CRL1505) strain. (**A**) Growth curve of the wild-type *L. rhamnosus* CRL1505 and Δ*mbf*CRL1505 strains. Lactobacilli were cultured in MRS broth for 24 h. OD_660_ was measured every 30 min. (**B**) Gram stain and scanning electron microscope (SEM) analysis of the wild-type *L. rhamnosus* CRL1505 and Δ*mbf*CRL1505 strains.

To study the effect of the *mbf* gene on the growth of *L. rhamnosus*, we measured the OD_660_ of the strains CRL1505 and Δ*mbf*CRL1505 in cultures of MRS broth for 24 h. No significant differences were observed between the WT and the mutant strains, indicating that the knockout of the *mbf* gene does not influence the growth of *L. rhamnosus* CRL1505 (Figure 3A). In fact, 5 × 10^9^ CFU/mL were detected at hour 24 for both Δ*mbf*CRL1505 and WT strains (data not shown).

We also studied the *L. rhamnosus* CRL1505 and Δ*mbf*CRL1505 cells by Gram staining and by SEM analysis. No significant differences were observed in the morphology of both strains with the methods used (Figure 3B).

### 2.3. L. rhamnosus *Δ*mbfCRL1505 Adhesion to Mucins and PIE Cells

**Figure 4:**
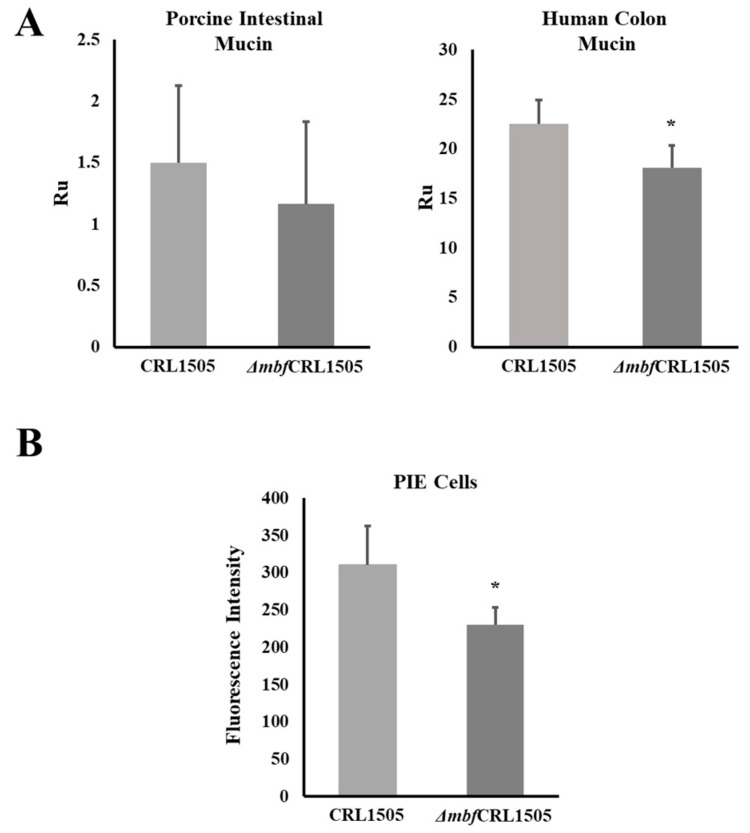
Adhesive capacities of the *mucus-binding factor* knockout *Lacticaseibacillus rhamnosus* CRL1505 *(*Δ*mbf*CRL1505) strain. (**A**) Adhesion of the wild-type *L. rhamnosus* CRL1505 and Δ*mbf*CRL1505 strains to porcine intestinal mucin and human colonic mucin. (**B**) Adhesion of the wild-type *L. rhamnosus* CRL1505 and Δ*mbf*CRL1505 strains to porcine intestinal epithelial (PIE) cells. The results represent data from three independent experiments. Asterisks indicate significant differences when compared to the wild-type strain (* *p* < 0.05).

The ability of the *L. rhamnosus* strains to adhere to soluble human colon mucin and soluble porcine ileal mucin was evaluated by the Biacore assay. The WT CRL1505 strain had the ability to adhere to both types of mucins as shown in Figure 4A. *L. rhamnosus* Δ*mbf*CRL1505 had a diminished capacity to adhere to the porcine mucin as shown by the resonance units that had values close to 1; however, this difference was not statistically significant when compared to the WT strain. In addition, the adhesiveness of the Δ*mbf*1505 strain to the human colonic mucin was significantly reduced when compared to the WT *L. rhamnosus* CRL1505 (Figure 4A). 

We also observed a difference when the adhesion of the WT CRL1505 and the Δ*mbf*CRL1505 strains to PIE was evaluated (Figure 4B). The adhesiveness of the Δ*mbf*1505 strain to PIE cells was significantly lower compared to the WT strain as shown by the difference in fluorescence units.

### 2.4. Immunomodulatory Activity of L. rhamnosus *Δ*mbfCRL1505 In Vivo

**Figure 5:**
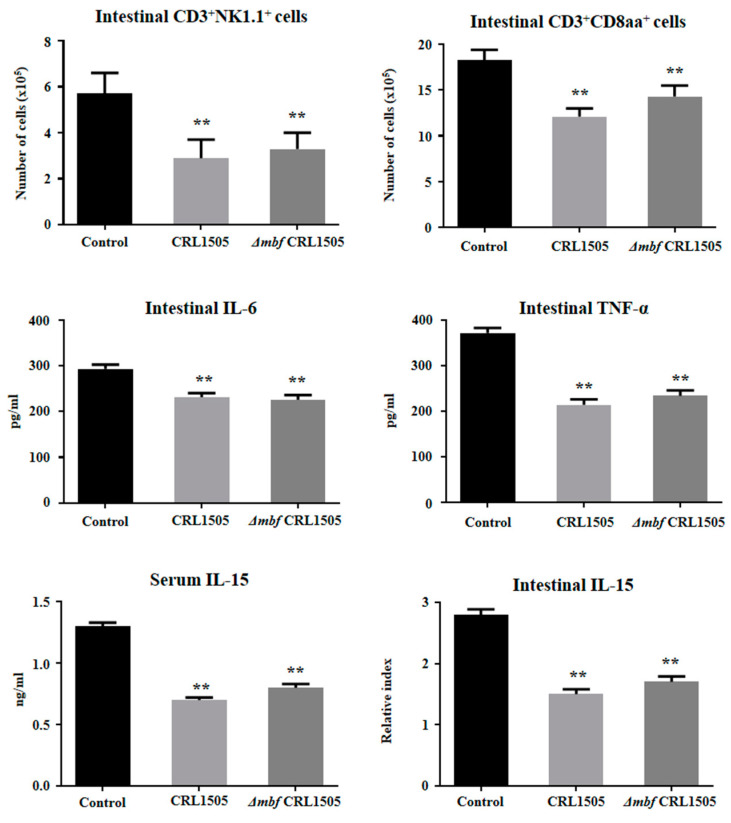
Immunomodulatory capacities of the *mucus-binding factor* knockout *Lacticaseibacillus rhamnosus* CRL1505 (Δ*mbf*CRL1505) strain on the intestinal innate antiviral immune response triggered by the activation of the Toll-like receptor 3 (TLR3). Balb/c mice (6-week-old) were orally treated with the wild-type *L. rhamnosus* CRL1505 or Δ*mbf*CRL1505 strains (10^8^ cells/mouse) for two consecutive days prior to the intraperitoneal injection of the TLR3 agonist poly(I:C). Untreated mice challenged with poly(I:C) were used as controls. The numbers of CD3^+^NK1.1^+^ and CD3^+^CD8αα^+^ intraepithelial lymphocytes (IELs), the levels of intestinal interferon (IFN)-β, IFN-γ, interleukin (IL)-6, tumor necrosis factor (TNF)-α and IL-15 and the level of serum IL-15 were determined 2 days after the poly(I:C) challenge. The results represent data from three independent experiments. Asterisks indicate significant differences when compared to the poly(I:C) control group (** *p* < 0.01).

We next aimed to evaluate whether the Δ*mbf*CRL1505 strain was able to modulate the mucosal inflammatory response and protect against the damage induced by TLR3 activation by using in vivo mice models. For that purpose, two sets of experiments were performed in 6-week-old Balb/c mice. First, *L. rhamnosus* CRL1505 or Δ*mbf*CRL1505 were orally administered to mice as described in materials and methods prior to the intraperitoneal injection of poly(I:C). The administration of poly(I:C) significantly increased the levels of intestinal pro-inflammatory cytokines and the recruitment of CD3^+^NK1.1^+^CD8αα^+^ intraepithelial lymphocytes (IELs) (Figure 5). The oral administration of *L. rhamnosus* Δ*mbf*CRL1505 significantly reduced the levels of intraepithelial lymphocytes (CD3^+^NK1.1^+^CD8αα^+^) and pro-inflammatory mediators (TNF-α, IL-6 and IL-15) in the intestinal mucosa when compared with the control mice (Figure 5). The Δ*mbf*CRL1505 strain also reduced the concentrations of IL-15 in serum. Of note, the concentrations of intestinal TNF-α, IL-6 and IL-15, serum IL-15 and the numbers of the intestinal CD3^+^NK1.1^+^CD8αα^+^ cells in Δ*mbf*CRL1505-treated mice were not different from those observed in the group of animals that received the WT CRL1505 strain before poly(I:C) challenge (Figure 5).

**Figure 6:**
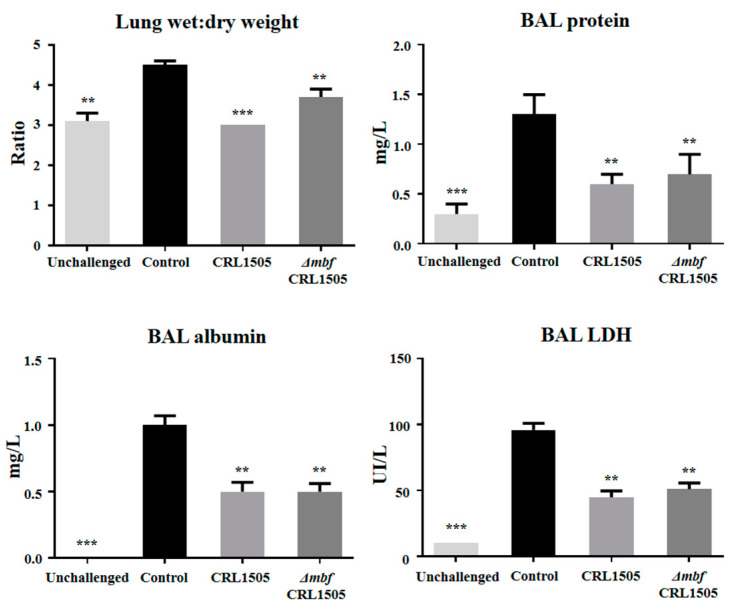
Immunomodulatory capacities of the *mucus-binding factor* knockout *Lacticaseibacillus rhamnosus* CRL1505 *(*Δ*mbf*CRL1505) strain on the respiratory innate antiviral immune response triggered by the activation of the Toll-like receptor 3 (TLR3). Balb/c mice (6-week-old) were orally treated with the wild-type *L. rhamnosus* CRL1505 or Δ*mbf*CRL1505 strains (10^8^ cells/mouse) for two consecutive days prior to the nasal administration of the TLR3 agonist poly(I:C). Untreated mice challenged with poly(I:C) were used as controls. Lung wet:dry weight and bronchoalveolar lavage (BAL) proteins, lactate dehydrogenase (LDH) and albumin were determined 2 days after the poly(I:C) challenge. The results represent data from three independent experiments. Asterisks indicate significant differences when compared to the poly(I:C) control group (** *p* < 0.01; *** *p* < 0.001).

**Figure 7:**
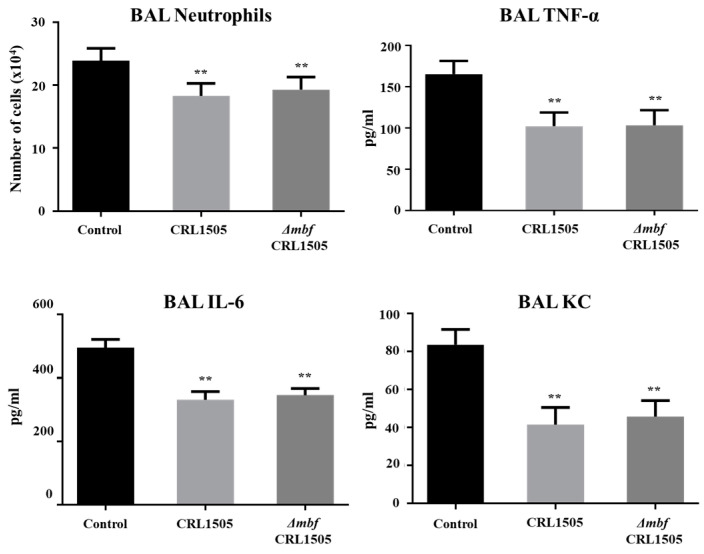
Immunomodulatory capacities of the *mucus-binding factor* knockout *Lacticaseibacillus rhamnosus* CRL1505 *(*Δ*mbf*CRL1505) strain on the respiratory innate antiviral immune response triggered by the activation of the Toll-like receptor 3 (TLR3). Balb/c mice (6-week-old) were orally treated with the wild-type *L. rhamnosus* CRL1505 or Δ*mbf*CRL1505 strains (10^8^ cells/mouse) for two consecutive days prior to the nasal administration of the TLR3 agonist poly(I:C). Untreated mice challenged with poly(I:C) were used as controls. The numbers of neutrophils, tumor necrosis factor (TNF)-α, interleukin (IL)-6, and IL-8 in bronchoalveolar lavage (BAL) samples were determined 2 days after the poly(I:C) challenge. The results represent data from three independent experiments. Asterisks indicate significant differences when compared to the poly(I:C) control group (** *p* < 0.01).

In the second set of experiments, the WT CRL1505 or Δ*mbf*CRL1505 strains were orally administered to different groups of mice, which then were nasally challenged with poly(I:C). As it was reported previously [14,15,16], the nasal stimulation with poly(I:C) significantly increased the levels of protein, albumin and LDH in BAL samples as well as the lung wet:dry weight, indicating the ability of the TLR3 agonist to induce lung inflammatory damage (Figure 6). The oral administration of *L. rhamnosus* Δ*mbf*CRL1505 significantly decreased the levels of the biochemical markers of lung tissue damage (Figure 6). In addition, Δ*mbf*CRL1505 treatment reduced the recruitment of neutrophils into the respiratory tract and the concentrations of TNF-α, IL-6 and KC in BAL samples when compared to control mice (Figure 7). Of note, the values of those parameters in mice receiving *L. rhamnosus* Δ*mbf*CRL1505 were not different from those observed in WT CRL1505-treated animals (Figure 7).

**Figure 8:**
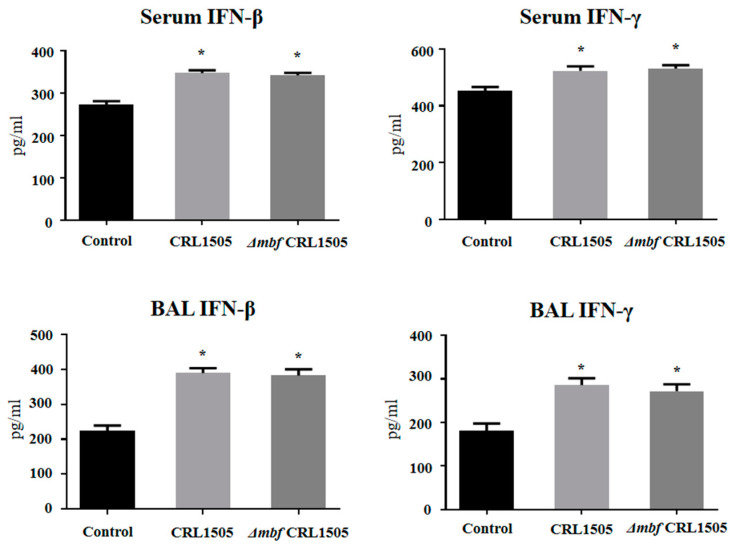
Immunomodulatory capacities of the *mucus-binding factor* knockout *Lacticaseibacillus rhamnosus* CRL1505 *(*Δ*mbf*CRL1505) strain on the respiratory innate antiviral immune response triggered by the activation of the Toll-like receptor 3 (TLR3). Balb/c mice (6-week-old) were orally treated with the wild-type *L. rhamnosus* CRL1505 or Δ*mbf*CRL1505 strains (10^8^ cells/mouse) for two consecutive days prior to the nasal administration of the TLR3 agonist poly(I:C). Untreated mice challenged with poly(I:C) were used as controls. The levels of serum and bronchoalveolar lavage (BAL) interferon (IFN)-β and IFN-γ were determined 2 days after the poly(I:C) challenge. The results represent data from three independent experiments. Asterisks indicate significant differences when compared to the poly(I:C) control group (* *p* < 0.05).

The concentrations of IFN-β and IFN-γ in BAL and serum were evaluated as shown in Figure 8. *L. rhamnosus* Δ*mbf*CRL1505 was able to significantly increase the levels of serum and respiratory IFN-β and IFN-γ when compared to controls. It was also observed that IFN-β and IFN-γ concentrations in Δ*mbf*CRL1505-treated mice were not different from those observed in the group of animals that received the WT CRL1505 strain before poly(I:C) challenge (Figure 8).

## 3. Discussion

In recent years, the use of microorganisms with the ability to modulate the immune system (immunobiotics) has notably increased because they are capable of preventing and reducing the severity of infections in humans and animals [17,18]. This is especially important given the need to find new tools that allow combating pathogens multi-resistant to antimicrobials, a global health problem which is constantly increasing [1]. In this sense. immunobiotics have been conclusively recognized as having beneficial effects on the mucosal innate and adaptive immune responses, and thus the ability to increase the resistance of the host against pathogens [19]. It is thought that the microbial associated molecular patterns (MAMPs) expressed in immunobiotic strains are effectively recognized by the host’s PRRs, activating signaling pathways that modulate the expression of various immune factors such as cytokines, chemokines, and adhesion molecules [20] that affect the strength and quality of immune responses [21].

It was generally concluded that probiotic bacteria had to adhere to the intestinal epithelium to effectively promote MAMP-PRR interaction and thus exert the immunomodulatory effect. In fact, host adhesion capacity is a classic selection criterion for candidate probiotic bacteria since colonization was thought to participate in strengthening the intestinal barrier, modulating metabolic functions, and regulating immune responses [22]. However, the relationship between the adhesion capacity of probiotics and immunomodulation is not clear and there contrasting studies in this regard [13]. In this work, we aimed to advance in the knowledge of the mechanisms by which the immunobiotic *L. rhamnosus* CRL1505 strain exert its immunomodulatory effects by studying the impact of adhesion to the intestinal mucosa through the *mbf* protein. Thus, using genetic engineering techniques we eliminated the mucus-binding factor from the CRL1505 genome, and demonstrated that this strain does not need this adhesion protein to carry out its immunomodulatory functions both at local (intestinal) and distant (respiratory) mucosal sites.

Among the proteins involved in the adhesion of *L. rhamnosus* to the intestinal mucosa is the *mbf* protein, a homolog of adhesin internalin J [4]. This protein contains four anchor repeats to the surface of the cell wall. This anchoring structure can be found in surface proteins of different microorganisms. For example, in *Listeria monocytogenes,* this protein accomplishes pleiotropic functions, including peptidoglycan metabolism, protein processing, adhesion to the mucosal surface and invasion to host tissue [23]. Furthermore, this anchoring repeat has been predominantly identified in lactobacilli that are naturally found in the intestine and promote cell adhesion to mucins [13]. Here, we successfully generated a *mbf*-deficient strain for *L. rhamnosus* CRL1505 by double homologous recombination. Western blot analysis confirmed that the expression of this protein was suppressed. Our results showing the absence of a 45 kDa band are in agreement with the results previously obtained for *L. rhamnosus* FSMM22 [13] and *L. rhamnosus* GG [4]. The *mbf* protein is a bacterial surface protein with a LPXTG motif located at the C-terminus of the protein and binds to peptidoglycan through its sortase activity. The threonine–glycine bond within this motif is cleaved and the threonine residue is covalently bound to the peptidoglycan, allowing it to adhere to the bacterial surface [24]. This structure suggests that the *mbf* protein is not involved in the organization of the cell wall; therefore, it has no effect on growth. In line with these findings, no significant differences were observed in the viability or growth of the Δ*mbf*CRL1505 strain compared to the WT CRL1505 strain in this study. Neither were differences observed in the cell surface of both strains by means of Gram staining or SEM analysis.

The immunomodulatory activities of *L. rhamnosus* CRL1505 have been extensively characterized by our research group [19]. We have demonstrated that the CRL1505 strain is capable of beneficially modulate the TLR3-mediated intestinal innate immune response and reduce the local inflammatory tissue damage after its oral administration [25]. *L. rhamnosus* CRL1505 can substantially modify the immunotranscriptomic response of intestinal epithelial cells after TLR3 activation, inducing an enhancement of type I IFNs and antiviral factors and a differential modulation of cytokines, chemokines, and adhesion molecules [26]. In vitro studies demonstrated that *L. rhamnosus* CRL1505 has the capability to increase IFN-β and IFN-γ production in poly(I:C)-challenged intestinal antigen-presenting cells [27,28]. We also demonstrated that the CRL1505 strain is able to increase IFNs and antiviral factors in an in vivo mouse model of TLR3-mediated intestinal inflammation. In addition, a reduction in TLR3-mediated intestinal tissue injury was observed when this immunobiotic strain was administered, an effect that was achieved through the modulation of IELs response [14]. Furthermore, we showed that the CRL1505 strain does not need to be alive in order to exert its immunomodulatory effect since bacterium-like particles obtained after heat and acid treatment improve the intestinal and systemic immune responses elicited by an attenuated rotavirus vaccine [12].

These previous results led us to speculate that the expression of adhesion factors is not a necessary condition for CRL1505 to exert its immunomodulatory effect. Then, with the aim of providing evidence to validate this hypothesis we performed comparative studies of the WT CRL1505 and Δ*mbf*CRL1505 strains. First, we demonstrated that the adherence of the Δ*mbf*CRL1505 strain to mucins and PIE cells is different to the observed for WT CRL1505. Our results indicate that in did, the *mbf* is associated to the adhesion capabilities of *L. rhamnosus* CRL1505. We also showed that both WT CRL1505 and Δ*mbf*CRL1505 are able to equally modulate the TLR3-mediated intestinal immune response, regulating the production of the pro-inflammatory cytokines IL-6, IL-15 and TNF-α as well as the levels of the CD3^+^NK1.1^+^CD8αα^+^ IELs, which were shown to mediate the intestinal inflammatory injury [29]. Our data agree with previous results obtained with *L. rhamnosus* GG in which the reduction in adhesion capacity did not affect its immunomodulatory activity in the intestinal mucosa. A 2-fold decrease in the adhesion capacity of the mutant CMPG5230 (lacking *MbaA*) to the murine gastrointestinal tract compared to WT *L. rhamnosus* GG was observed. However, preincubation of intestinal epithelial cells with the WT GG strain and the mutant CMPG5230 were equally effective in reducing IL-8 and TNF-α expression in response to *Salmonella* infection [6].

Orally administered *L. rhamnosus* CRL1505 has the ability to beneficially modulate the respiratory innate immune response triggered by TLR3 activation [10] increasing the resistance against respiratory syncytial virus [30] and influenza virus [31] infections. The oral administration of the CRL1505 strain stimulates the Th1 response in the intestinal mucosa inducing the mobilization CD4^+^IFN-γ^+^ T cells from the intestine to the lungs. IFN-γ-producing T cells in the respiratory tract modulate the innate immune responses through their ability to impact on CD11c^+^SiglecF^+^MHC-II^hi^ alveolar macrophages function [32]. Then, mice orally treated with the CRL1505 strain have reduced production of TNF-α, IL-6 and KC and diminished recruitment of inflammatory cells after poly(I:C) challenge, which correlated with lower inflammatory lung damage. Considering that the beneficial effect induced by *L. rhamnosus* CRL1505 in the respiratory tract is related to its ability to impact on intestinal immunity, we also evaluated here whether adhesion mediated by the *mbf* protein impacted on its capacity to modulate the respiratory antiviral immunity. As expected, we observed that orally administered WT CRL1505 and Δ*mbf*CRL1505 are equally effective in modulating the TLR3-triggered respiratory immune response.

It was reported that *L. rhamnosus* GG possess adhesins for mucins and intestinal epithelial cells, including fimbriae and MabA, and that the *mbf* proteins are considered to have auxiliary functions [6]. However, Nishiyama et al. demonstrated that the *mbf* protein significantly contributes to its adherence to the extracellular matrix [13]. It was also shown that the function of the *mbf* protein depends on the strain of lactobacillus under study. Consequently, while *mbf* probably represents one of the key mucosal adhesins on the cell surface of the *L. rhamnosus* LC705 strain, this surface-localized protein represents only a small fraction of the total mucus-binding capacity in *L. rhamnosus* GG [4]. Our results showed that the Δ*mbf*CRL1505 strain was able to adhere to both mucins and PIE cells although with a significant reduced capacity compared to the WT CRL1505 strain. Thus, other factors could contribute to the adhesion of the immunobiotic CRL1505 strain to the intestinal mucosa. Analyzing those factors in the genome of *L. rhamnosus* CRL1505, obtaining mutant strains, and evaluating their immunomodulatory activities are studies that should be carried out in the future to complement the work presented here.

## 4. Materials and Methods

### 4.1. Bacterial Strains and Growth Conditions

*L. rhamnosus* CRL1505 was obtained from the culture collection of CERELA-CONICET (Tucumán, Argentina). *Lactococcus lactis* IL1403, which carries a temperature-sensitive integration vector pSG^+^E2 was also used. Bacteria (10^10^ CFU stored at −70 °C) were activated and cultured for 12 h at 37 °C (final log phase) in Man-Rogosa-Sharpe (MRS) broth culture media. This medium was supplemented with 25 μg/mL erythromycin (Em) for plasmid selection when necessary.

### 4.2. Construction of mbf Knockout Gene by PCR

Total DNA from *L. rhamnosus* CRL1505 was extracted using the DNeasy Blood & Tissue Kit (QIAGEN, Hilden, Germany) according to the manufacturer’s instructions and it was used as a template to run the PCR reaction with the combination of primers Lr13—Lr14 and the PrimeSTAR^®^ Max DNA Polymerase (Takara Bio, Kusatsu, Japan) using the corresponding annealing temperatures (Table 1). The primers used in this study were chemically synthesized by Eurofin Genomics Corporation (Tokyo, Japan) (Table 1). A second nested PCR was performed using a combination of primers Lr3—Lr4 (5′ upstream) and Lr5—Lr6 (3’ downstream) (Table 1). The specific PCR products were isolated with the commercial Nucleo Spin Gel and PCR Clean-up Kit (MACHEREY-NAGEL, Düren, Germany) according to the manufacturer’s instructions. 

Finally, an over RAP-PCR was performed using the two fragments obtained in the second PCR as templates, and the primers Lr3 and Lr6 (Table 1) to prepare fragments for the *mbf* knockout. Fragments for *mbf* knockout were isolated and a restriction enzyme treatment (37 °C, 1 h) was performed with *Sac*I and *Sal*I (New England Biolabs, Ipswich, MA, USA). The fragment treated with the restriction enzymes was cut out from an agarose gel (1.2%) and purified with the commercial kits.

### 4.3. Recombinant Cloning of the mbf Knockout Gene and Construction of the Knockout Strain

The amplified fragment was inserted into the thermosensitive vector pSG^+^E2 using DNA Ligation Kit Version 1 (Takara Bio Inc., Kusatsu, Japan) and the new plasmid was designed as pSG^+^*mbf*. The pSG^+^*mbf* plasmid was introduced into *L. rhamnosus* CRL1505 competent cells and transformed by electroporation as previously described [33]. The obtained colonies were subjected to colony PCR using primers p119 and p120 (Table 1), and the amplified sample was confirmed by agarose gel electrophoresis.

A double-crossover recombination was performed next. For the first recombination, the CRL1505 strain containing the plasmid pSG^+^*mbf* was incubated during 2 days in MRS with Em at 42 °C, since at this temperature the vector cannot replicate and is integrated into the genome. For the second recombination, the primary recombinants were incubated during 2 days in MRS at 30 °C, where the Em resistance gene is shed, and secondary recombination occurs. The knockout DNA was extracted using the DNeasy^®^ Blood Tissue Kit (250) (QIAGEN). Then, a PCR was performed using primers Lr17 and Lr18 (Table 1). This was gel-purified, and the bands of WT and recombinant strains were compared by electrophoresis. The *mbf* knockout strain was designated as Δ*mbf*CRL1505.

### 4.4. Protein Detection by Western Blotting

Surface proteins from the CRL1505 and Δ*mbf*CRL1505 strains were extracted as previously described [34]. Protein concentration was determined with BCA protein Assay Kit (Thermo Fisher SCIENTIFIC, Tokyo, Japan) following the manufacturer’s instructions.

Surface proteins were analyzed by SDS-PAGE followed by staining with Coomassie brilliant blue and verified by Western blotting using a specific antibody to the *mucus-binding factor* of *L. rhamnosus* (rabbit anti CRYVRLAADSAAASGTFPKD provided by Prof. Keita Nishiyama). 

### 4.5. Effect of the Knockout mbf Gene on the Viability and Phenotype of L. rhamnosus CRL1505

*L rhamnosus* CRL1505 and Δ*mbf*CRL1505 were cultured as described above. To evaluate the difference in the growth of both strains, 2% of the preculture was seeded on MRS, cultured at 37 °C, 60 rpm, and the bacterial growth was measured with TVS062CA (ADVANTEC^®^, Dublin, CA, USA) at OD_660_ every half hour for 24 h. In addition, bacterial cell counts were performed at hour 24 using MRS agar plates.

In order to evaluate the effect of the knockout gene on the phenotype of the Δ*mbf*CRL1505 strain, a Gram stain was performed (Muto Pure Chemicals Co., Ltd., Tokyo, Japan). In addition, a scanning electron microscope analysis was performed. Bacteria culture was diluted 10-fold with PBS, and this was added dropwise to a membrane filter, polycarbonate, 0.2 µm × 13 mm (ADVANTEC^®^). This filter was allowed to stand in 2% glutaraldehyde for 1 h in RT to fix the cells. Then, dehydration was carried out stepwise with 50, 60, 70, 80, 90 and 99.5% ethanol for 20 min each. Finally, samples were immersed in t-butyl alcohol, lyophilized, and treated with platinum palladium and these were examined with a KEYENCE VE-9800 scanning electron microscope.

### 4.6. Binding Ability Assays

The adhesion ability of the *L. rhamnosus* CRL1505 and Δ*mbf*CRL1505 strains to soluble human colon mucin (sHCM) was obtained from Tohoku University Graduate School of Medicine under the approvement by the ethics committee of Tohoku University Graduate School of Medicine and soluble porcine ileal mucin (sPIM) were prepared according to the method in our previous study [35]. Mucin was used in an examination by the principle of surface plasmon resonance. For this purpose, Biacore experiments were performed using a Biacore 1000 (GE Healthcare Bio-Sciences K.K., Sheffield, UK) at 25 °C in HBS-EP buffer as previously described [35]. Culture cells were washed twice with PBS and then they were lyophilized. Next, 1 mg of lyophilized cells was suspended in 1 mL of HBS-EP buffer. This analysis solution (15 µL) was flowed through the ligand immobilized on the CM5 sensor chip (GE Healthcare Bio-Sciences K.K.). The amount of adhesion was defined as the Resonance Unit (RU) value 200 s after injection of the analysis solution minus the baseline RU value.

The binding ability of the WT CRL1505 and the Δ*mbf*CRL1505 strains to porcine intestinal epithelial (PIE) cells was studied by CFDA fluorescence measurement. CFDA (5-Carboxyfluorescein Diacetate, Invitrogen, Waltham, MA, USA) is a carboxyfluorescein dictate succinimidyl ester that is activated inside the bacterial cells by cellular esterases. After washing the cultures of WT CRL1505 and the Δ*mbf*CRL1505 strains twice with PBS, they were suspended in 5 mL of PBS and 50 µL of 1 mM CFDA, cultured at 37 °C for 1 h under light-shielded conditions and collected by centrifugation (8000 rpm, 10 min, 4 °C).

PIE cells were seeded on a collagen (Type I) coated 12-well plate (SUMILON) at 5000 cells/well, and then pre-cultured for 3 days at 37 °C with 5% CO_2_. Confluent cells were washed 3 times with PBS. Then, 5 × 10^7^ fluorescent bacterial cells/mL were added and incubated at 37 °C, 5% CO_2_ and light-shielded conditions for 48 h. After washing with PBS, 100 µL of 1% SDS and 0.1 M NaOH solution were added, and the cells were lysed at 60 °C for 1 h under light-shielding conditions. The fluorescence was measured with a microplate reader (Perkin Elmer, Fukuoka, Japan).

### 4.7. Animals, Feeding Procedures and Administration of Poly(I:C)

Mice (6-week-old Balb/c) were obtained from the closed colony kept at CERELA-CONICET (Tucumán, Argentina). Animals were housed in plastic cages in a controlled atmosphere with a 12 h light/dark cycle. Parameters were studied in 5 mice per group for each time point. All groups were fed a conventional balanced diet ad libitum. All experiments were carried out according to the Guide for Care and Use of Laboratory Animals and approved by the Ethical Committee of Animal Care at CERELA-CONICET under the BIOT-CRL/19 protocol, and all efforts were made to minimize suffering. No signs of discomfort or pain and no deaths were observed before mice reached the endpoints.

Two sets of in vivo experiments were performed in mice to evaluate the immunomodulatory activities of Δ*mbf*CRL1505. (A) The WT CRL1505 or Δ*mbf*CRL1505 strains (10^8^ cells/mouse) were orally administered for two consecutive days prior to the intraperitoneal injection with 100 µL of PBS containing 30 µg of the TLR3 agonist poly(I:C). Bacteria were administered intragastrically. Untreated control mice were challenged with poly (I:C) similarly. (B) The WT CRL1505 or Δ*mbf*CRL1505 strains (10^8^ cells/mouse) were orally administered for two consecutive days. On the third day, treated mice were nasally challenged with poly(I:C) (250 μg/mouse) for three consecutive days. Untreated control mice were challenged with poly(I:C) similarly. In both set of experiments, the innate antiviral immune response was evaluated 2 days after poly(I:C) stimulation.

### 4.8. Serum, Bronchoalveolar Lavage (BAL) and Intestinal Fluid Sampling

Blood samples were obtained from xylazine and ketamine-anesthetized animals through cardiac puncture at the end of each treatment and were collected in tubes containing EDTA as an anticoagulant. BAL fluid was collected from each animal via cannulation of the exposed trachea and gentle flushing of the lungs with 0.5 mL of sterile PBS. The recovered fluid was centrifuged at 900× *g* for 10 min and the supernatant fluids were frozen at −70 °C until use.

Intestinal fluid samples were obtained as the method described previously [36]. Briefly, the small intestine was washed with 5 mL of PBS and the resultant fluid was centrifuged at 10,000× *g* during 10 min to separate particulate material. The supernatant was kept frozen until use. 

### 4.9. Cytokines and Chemokines Analysis

Tumor necrosis factor (TNF)-α, interferon (IFN)-γ, IFN-β, IL-6, IL-8, IL-10 and IL-15 concentrations in serum, BAL and intestinal fluid samples were measured with commercially available enzyme-linked immunosorbent assay (ELISA) technique kits following the manufacturer’s recommendations (R&D Systems, Minneapolis, MN, USA).

### 4.10. Determination of Blood Cell Populations

Blood samples were obtained as described above. Total number of leukocytes was determined using a hemocytometer. Differential cell counts in blood were obtained by microscopically counting 200 cells in smears stained with May Grunwald-Giemsa as described before [37].

### 4.11. Biochemical Assay of BAL Samples

Protein and albumin content are a measure to quantitate increased permeability of the bronchoalveolar–capillarity barrier, and lactate dehydrogenase (LDH) activity is an indicator of general cytotoxicity. Those parameters were determined in the BAL fluid. Protein content was measured by the bicinchoninic (BCA) protein assay (Pierce Biotechnology Inc., Rockford, IL, USA). Albumin content was determined colorimetry based on albumin binding to bromcresol green using an albumin diagnostic kit (Wiener Lab, Buenos Aires, Argentina). Results were expressed in milligrams per liter of BAL fluid. LDH activity was determined by measuring the formation of the reduced form of nicotinamide adenine dinucleotide (NAD) using commercial reagents and procedures (Wiener Lab). Results were expressed as units per liter of BAL fluid.

### 4.12. Lung Wet:Dry Weight Ratio

Mice were euthanized and exsanguinated. Their lungs were removed, weighed, and dried in an oven at 55 °C for 7 days. After drying, the lungs were weighed again. Wet:dry weight ratio was then calculated as an index of intrapulmonary fluid accumulation, without correction for blood content.

### 4.13. Study of Intestinal Intraepithelial Lymphocytes

IELs were isolated as previously described [14]. Briefly, Peyer’s patches were removed, the small intestine was opened longitudinally and cut into 5 mm long pieces. Samples were washed twice in PBS containing 150 µg/mL streptomycin and 120 U/mL penicillin. The pieces were then stirred at 37 °C in prewarmed RPMI 1640 containing 150 µg/mL streptomycin, 120 U/mL penicillin, and 5% FCS for 30 min, followed by vigorous shaking for 40 s. This process was repeated. The supernatants were passed through a small cotton-glass wool column to remove cell debris and then, they were separated on a Percoll density gradient (Amersham Biosciences, Amersham, UK). A discontinuous density gradient (40 and 70%) was used. The cells that layered between the 40 and 70% fractions were collected as IELs. These IELs contained >90% CD3^+^ cells as determined by FACS analysis. Cellular phenotypes in IELs populations were analyzed by flow cytometry using FITC-conjugated anti-CD3, and PE-conjugated anti-NK1.1 (PK136), and anti-CD8a (CTCD8b), (R&D Systems). To prevent non-specific binding, respective isotype Abs were used as controls. Images of labeled cells were acquired on a BD FACSCaliburTM flow cytometer (BD Biosciences, East Rutherford, NJ, USA) and analyzed with FlowJo software (TreeStar, Woodburn, OR, USA).

### 4.14. Statistical Analysis

Experiments were performed in triplicate and results were expressed as the mean ± standard deviation (SD). After verification of the normal distribution of data, 2-way ANOVA was used. Tukey’s test (for pairwise comparisons of the means) was used to test for differences between the groups. Differences were considered significant at *p* < 0.05.

## 5. Conclusions

Although more studies must be carried out to determine the exact bacterial molecules from *L. rhamnosus* CRL1505 responsible for its immunomodulatory capacities, the results of this work indicate that the *mbf* protein is not involved in the immunobiotic effects induced by this strain. The adherence mediated by the *mbf* protein is not a necessary condition for *L. rhamnosus* CRL1505 to improve innate immunity and protect against inflammatory damage, both in the intestinal and the respiratory tracts. This study is an important step towards understanding the mode of action of immunobiotics and the relationship between adhesion capacity and their immunomodulatory effects.

## Figures and Tables

**Table 1:** Sequences of the primers used in this study.

Primer	Sequence (5′—3′)	Restriction Site	Annealing Temperature
**Lr13**	GTCAACCATCTAGAAGCATGAA		55 °C
**Lr14**	TACTGCGATTCATCGCTAGG		55 °C
**Lr3**	ACACGTCGTCGAACAGTTGTGGTGAAGTGTGTTGAT	*Sal*I	55 °C
**Lr4**	CAACCATCACTGGCGGCCAGCCTCATCCAC		55 °C
**Lr5**	GTGGATGAGGCTGGCCGCCAGTGATGGTTG		55 °C
**Lr6**	ACACGAGCTCCCGCCAGTGATTAAACTGGT	*Sac*I	55 °C
**p119**	CTTTTACGTTTCCGCCATTC		50 °C
**p120**	ATTTCATCAATGGCCTCAGT		50 °C
**Lr17**	GGTTCGTATACTGCCGTGCCA		50 °C
**Lr18**	GCTTGTTGACCAGGCTGATATTCT		50 °C

## Data Availability

All data generated and analyzed during this study are included in this published article.
